# Physiological reconstruction of blood glucose level using CGMS-signals only

**DOI:** 10.1038/s41598-022-09884-5

**Published:** 2022-04-06

**Authors:** Tomas Koutny

**Affiliations:** Department of Computer Science and Engineering, NTIS – New Technologies for the Information Society, Faculty of Applied Sciences, University of West Bohemia, Plzeň, 306 14 Czech Republic

**Keywords:** Diagnostic markers, Diabetes

## Abstract

Patient with diabetes must regularly monitor blood glucose level. Drawing a blood sample is a painful and discomfort experience. Alternatively, the patient measures interstitial fluid glucose level with a sensor installed in subcutaneous tissue. Then, a model of glucose dynamics calculates blood glucose level from the sensor-measured, i.e., interstitial fluid glucose level of subcutaneous tissue. Interstitial fluid glucose level can significantly differ from blood glucose level. The sensor is either factory-calibrated, or the patient calibrates the sensor periodically by drawing blood samples, when glucose levels of both compartments are steady. In both cases, the sensor lifetime is limited up to 14 days. This is the present state of the art. With a physiological model, we would like to prolong the sensor lifetime with an adaptive approach, while requiring no additional blood sample. Prolonging sensor’s lifetime, while reducing the associated discomfort, would considerably improve patient’s quality of life. We demonstrate that it is possible to determine personalized model parameters from multiple CGMS-signals only, using an animal experiment with a hyperglycemic clamp. The experimenter injected separate glucose and insulin boluses to trigger rapid changes, on which we evaluated the ability to react to non-steady glucose levels in different compartments. With the proposed model, 70%, 80% and 95% of the calculated blood glucose levels had relative error less than or equal to 21.9%, 32.5% and 43.6% respectively. Without the model, accuracy of the sensor-estimated blood glucose level decreased to 39.4%, 49.9% and 99.0% relative errors. This confirms feasibility of the proposed method.

## Introduction

Diabetes is a heterogeneous group of disorders, which manifest with elevated blood glucose level (BG)^1^. Over the long term, elevated BG damages multiple organs, eventually leading to their failure. Therefore, a patient with diabetes must monitor BG to maintain it within normal range. To measure current BG, the patient has to draw a blood sample. It is a painful experience. It causes important discomfort to the patient. As a result, frequent BG sampling is not feasible, especially over the long-term^2^. A patient is willing to take 3–4 reference BG samples per day^3^.

There is Continuous Glucose Monitoring System (CGMS). Commonly, CGMS reports glucose level every 5 min. It has a sensor needle installed in subcutaneous tissue. With the needle, it continuously measures electric current, produced by a chemical reaction with glucose. Using a model of glucose dynamics, CGMS converts the measured current to a BG estimate that we denote as IG. CGMS is minimally invasive system with two issues.

The first issue is that IG can differ from BG significantly. Primarily, glucose propagates from blood to interstitial fluid across capillary wall. In the interstitial space, cells utilize part of the glucose, while the other part of the glucose leaves this space. As extracellular fluids continually mix together, BG and IG tend to equalize over the time^4^. With no significant change in BG magnitude, IG converges towards BG as they equalize. During meal, increased physical activity, stress of illness, etc., BG can change significantly. Then, IG reacts with a delay and both levels exhibit different magnitude and different rate of change. It is important to manage such events as they may lead to acute complications such as hypo- and hyperglycemic shock. In addition, continuous BG and IG patterns can provide a detail insight on the progress of the disease by showing how a specific patient reacts to particular BG disturbances. This opens a possibility to adjust drug dosage with increased accuracy. Therefore, we calculate continuous BG signal from the continuously measured IG signal by using a model of glucose dynamics^5^.

The second issue is sensor calibration. The calibration is a process of determining parameters, which CGMS use to convert the measured electric current to glucose level.

As the historically first option, a patient must periodically draw a blood sample to determine reference BG to calibrate CGMS. CGMS pairs the reference BG measurements with the electric-current measurements at respective times. Using these pairs, CGMS calculates the calibration parameters. To perform a successful calibration, a patient with diabetes must draw the reference BG sample when BG is steady and thus BG and IG can be considered as equal^6,7^. Improper calibration results to erroneous processing of the measured electric current, thus leading to wrong, reported glucose levels and subsequently raising patient’s disappointment with the CGMS therapy. As a result, the patient may deny the CGMS further, despite its benefits, when calibrated correctly.

As an alternative option, there is factory calibration^8,9^. In such a case, the calibration parameters are pre-determined, based on the manufacturing process and previously measured data. As a result, the patient does not calibrate the sensor during its lifetime. According to study^10^, sensors indicated for a period longer than 14 days would still likely require a calibration algorithm.

With both options, patient’s body works towards eliminating the CGMS sensor as it considers the sensor as a foreign element. During this process, measurement error increases. Eventually, patient’s body wins and the sensor no longer provides correct readings. Therefore, sensor lifetime spans up to 14 days, because the sensor’s internal logic is no longer able to recover from the physiologically induced measurement error. After that, the sensor must be physically replaced.

### Study rationale

Diabetes is public health problem. According to International Diabetes Federation, 415 million people are living with diabetes and 12% of global health expenditure is spent on diabetes^11^. Quality of life of a patient with diabetes would improve with CGMS that increases accuracy of calculated BG, reduces the associated pain and discomfort^12^, while significantly prolonging the sensor lifetime. To prolong the lifetime, one approach is to deliver an anti-inflammatory agent to reduce the body’s immune reaction^13^. Our approach aims to reduce the pain and discomfort by an improved processing of measured signals, while being compatible with the existing approaches to maximize the overall effectiveness. The key improvement is that our approach is adaptive. By computing personalized parameters of a glucose-dynamics model, it would continuously adapt to recent body changes and metabolic processes of an individual patient.

## Materials and methods

### Experimental setup

In this study, we conducted no experiment on a living animal. We reused measured data of a previously conducted experimental setup with hyperglycemic clamp on hereditary hypertriglyceridemic (hHTg) rats. The experimental data were provided by the Diabetology Center, University Hospital in Pilsen, Charles University in Prague. The respective committee of these institutions approved all the experimental protocols. All methods were carried out in accordance with relevant guidelines and regulations. The protocols and methods were approved before the ARRIVE guidelines^14^ came into the effect. Nevertheless, we checked its items to provide all the applicable information.

To process the measured data, we used our own specialized software that eventually evolved to SmartCGMS – continuous glucose monitoring and controlling framework^15^. This software enables us to conduct advanced research on diabetes treatment. The demanding nature of developing such a specialized software caused the delay from conducting the experiment to devising the proposed method.

hHTg rats were bred from Wistar rats to study metabolic abnormalities. This makes them suitable for diabetes-related experiments as they have disturbances in glucose metabolism^16^. hHTg rat displays hypertriglyceridemia, impaired glucose tolerance, hyperinsulinemia and insulin resistance. Impaired insulin action is responsible for the defective glucoregulation in this strain^17^.

Specifically, we reused results collected with six animals^19^, which had multiple sensors installed and each sensor successfully calibrated at the beginning of the experiment. All rats were male with respective weights of 418, 367, 360, 420, 430 and 360 g. At the same time, each animal had three Enlite CGMS sensors (Medtronic Diabetes, Northridge, CA) installed in subcutaneous tissue, skeletal muscle and subcutaneous adipose tissue, i.e., visceral fat, to provide three, different IG signals. To measure BG, we sampled arterial blood. Studies^18,19^ give further details on this experimental setup with a hyperglycemic clamp. In this study, we verify the possibility of completely removing the reference BG from the calibration process. Specifically, we demonstrate that magnitudes and delays of different IG signals contain enough information to reconstruct BG without needing any reference BG to determine personalized parameters of our glucose-dynamics model.

### Model of glucose dynamics

In previous studies, we established a model of glucose dynamics to calculate BG^18^ Eqs. (1) and (2) describe the model. Studies^5,18–20^ describe development of the model, including development of methods needed to determine its parameters. Initially, we tested the model with hereditary hHTg rats, using hyperglycemic clamp as the experimental study^19^. Then, we verified the model with humans^5^.

In Eqs. (1)–(3), *b(t)* and *i(t)* give BG and IG at respective time *t*. Equation (2) relates BG and IG at time *t* with future IG at time *φ*(*t)* – given by Eq. (1). Equation (1) comprises static, *Δt*, and dynamic component. With *h*-long interval, the dynamic component converts the observed IG change to a time-varying offset to account effect of the concentration-gradient rate of change. The *k*-parameter quantifies this effect, which affects IG over the *h*-long interval^5^.

As described in study^18^, the *p*-parameter expresses glucose gain from blood due to the structure of the capillary wall. The *cg*-parameter further limits this gain by applying the inter-compartment gradient of glucose levels across the capillary wall. The *c*-parameter covers difference between the inter-compartment glucose flux and glucose flux into cells from the interstitial fluid. Study^20^ gives further details.

1 $$\varphi \left( t \right) = t + \Delta t + \left\{ {\begin{array}{*{20}l} {k \times \frac{{i\left( t \right) - i\left( {t - h} \right)}}{h},} \hfill & { h \ne 0} \hfill \\ {0,} \hfill & {h = 0} \hfill \\ \end{array} } \right.$$

2 $$p\times b\left(t\right)+cg\times b\left(t\right)\times \left[b\left(t\right)-i\left(t\right)\right]+c=i(\varphi \left(t\right))$$

3 $$p\times b\left(t\right)+cg\times b\left(t\right)\times \left[b\left(t\right)-i\left(t+{\Delta t}_{m}\right)\right]+c=i(t+{\Delta t}_{f})$$

To make the calibration process independent on BG measurements, let us calculate BG using IG signals measured in different compartments. Accordingly, we transformed Eq. (2) to Eq. (3) to cover a physiological delay of glucose propagation from arterial blood.

In this study, we retained the *p, cg, c* and *Δt*_*f*_ (the original *Δt*) parameters in the modified model – Eq. (3). *Δt*’s *f* -subscript stands for future. We eliminated the *k* and *h* parameters, because (1) these parameters equaled zero for majority of human patients with diabetes^5^ and (2) we desired to avoid a possible overfitting of the model. Therefore, we have not implemented any sensor-error model at this stage of the presented research as it would increase the number of parameters.

As each compartment exhibits a different IG delay from arterial BG due to physiological reasons, we only added the *Δt*_*m*_ parameter to represent this delay. Hence, Eq. (3) relates BG at time *t* and IG at time *t* + *Δt*_*m*_ with future IG at time *t* + *Δt*_*f*_. *Δt*’s *m*-subscript stands for measured. Both *Δt* parameters account biological and technological delays, given by a construction of the particular sensor.

In study^5^ with human patients, we demonstrated that BG calculation with a priori determined parameters of Eqs. (1) and (2) improve accuracy over measured IG. With *Δt*_*m*_ = 0, the demonstration still holds as Eq. (3) reverts to the original model then.

In study^20^, we applied the Eq. (1) with *k* = 0 and *h* = 0 to glucose levels of subcutaneous tissue, skeletal muscle tissue and visceral fat. The glucose-dynamics model exhibited feasible error with all compartments and agreed with physiological findings of different studies. Skeletal muscle tissue and visceral fat exhibited greater metabolic activity than subcutaneous tissue, which produced less computational error.

**Statement #1**

As a priori determined parameters improve accuracy, average of multiple calculated BG signals improves the accuracy further.

Contrary to *in-vitro* and *in-silico* experimental setups, *in-vivo* setup cannot reproduce the same IG measurements due to the impossibility of establishing identical starting conditions. Therefore, we measured IG in different compartments at the same time. By applying Eq. (3) to these signals, we calculated arterial BG^18^. Because precision of the calculated BG affects its accuracy, we calculated multiple BGs from different compartments to determine their average, eventually.

When conducting study^5^, we determined parameters of “average patient with diabetes” on a completely different, human dataset. In this study, we reused the *p*, *cg* and *c* parameters from study^5^ as the a priori determined parameters, while placing *Δt*_*m*_ = 0. Table 1 gives these parameters, in the Scenario a) column. As this is an animal study, parameters of a different study eliminates a possible bias that would otherwise arise, if we would a priori determine the parameters from the animal subjects on which we are testing the proposed method.

**Table 1:** Parameters of glucose-dynamics model with respect to Table 2.

Parameters		A priori determined, parameters – Scenario a)	IG-only Statement #2-personalized parameters – Scenario c)	IG-only Statement #3-personalized parameters – Scenario d)	BG-personalized parameters
Name	Percentile				
*p* (unitless)	25th	1.046	0.897	0.889	0.794
	Median		1.156	1.003	0.987
	75th		1.409	1.457	1.129
*cg* [L/mmol]	25th	− 0.009	− 0.073	− 0.074	− 0.053
	Median		− 0.060	− 0.058	− 0.041
	75th		− 0.032	− 0.035	− 0.026
*c* [mmol/L]	25th	0.226	− 2.183	− 2.003	− 0.143
	Median		− 0.237	0.067	0.579
	75th		0.989	0.575	1.312
*Δt*_*m*_	25th	00:00	00:18	3:20	00:00
	Median		11:26	11:42	03:27
	75th		15:48	21:36	22:59
*Δt*_*f*_	25th	18:52	19:04	16:47	11:20
	Median		22:11	21:19	20:29
	75th		33:50	37:04	32:32

Median, 25th and 75th percentiles illustrate shape of the distribution.

**Statement #2**

As Statement #1 leads to correct BG calculation, it is possible to determine personalized model parameters for each compartment, where we measure IG.

To determine the personalized model parameters per compartment, we used average-calculated BG as the reference BG. Calculated BG comprises true, measured BG and time-varying error noise due to various technological and biological reasons. When determining model parameters, we attempt to reduce the error noise. A metric quantifies this noise with a scalar, by evaluating the difference between the referenced and calculated signals. When calculating BG from multiple IG signals, accordingly to Statement #1, let us refer to the sum of the metric scalars of all compartments as to the total error.

**Statement #3**

Repeated application of Statements #1 and #2 iteratively improves precision and accuracy of calculated BG, calculated from multiple IG signals of different compartments.

Repeated application of Statement #2 produced a sequence of total-error scalars. A scalar, which represented least total error, identified the best-known agreement of multiple model parameters (used to calculate the averaged BG from multiple IG signals).

Let us consider a single model-parameter identification step, by requiring that calculated BG per each compartment agree with calculated BG of the other compartments. For example, *p* = 0, *cg* = 0 and *c* = 0 parameters would satisfy this condition, but the calculated BG would not be physiologically valid. Therefore, we applied the iterative approach that avoids this adverse phenomenon without the need to define additional constraints on the model parameters, which would further increase the computational complexity.

## Results

To verify Statements #1 – #3, we analyzed the following scenarios:

- Scenario a) verifies default parameters
- Scenario b) verifies Statement #1
- Scenario c) verifies Statements #1 and #2
- Scenario d) verifies Statements #1, #2 and #3 – i.e., the output, calculated BG

**Figure 1:**
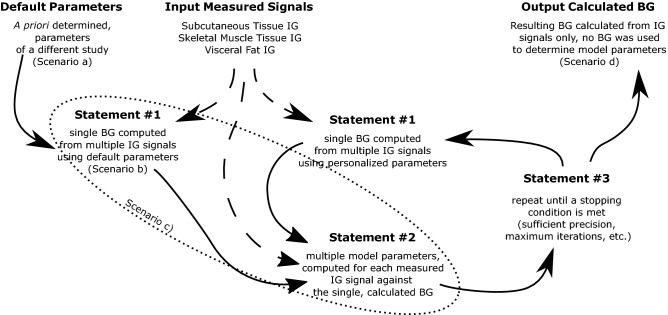
Calculation flow of the proposed method with depicted scenarios.

Figure 1 depicts the calculation flow and the scenarios. Solid, oriented curves present the calculation flow from the a priori determined parameters to the final, calculated BG. Dashed, oriented curves indicate, which calculation steps needed the input IG signals. The scenarios are associated with respective calculation steps. To quantify the fitness of the calculated BG, we used relative error. It is absolute difference between measured and calculated level, divided by the measured level.

**Scenario a)**

Using parameters of study^5^ as the a priori determined parameters, we calculated BG per individual compartment. The calculated BG exhibited less error than the IG signal, when compared to the measured BG.

- See Table 2, the "A priori determined parameters – Scenario a)" column.

**Table 2:** Calculated BG per individual IG signal.

Cumulative probability of less than or equal relative error	Relative error (and its improvement over IG that is considered as BG)			
	A priori determined parameters – Scenario a)	IG-only Statement #2- personalized parameters – Scenario c)	IG-only Statement #3- personalized parameters – Scenario d)	BG-personalized parameters
10%	3.7% (− 0.3%)	2.8% (+ 0.6%)	2.7% (+ 0.7%)	0.4% (+ 3.0%)
20%	7.8% (− 0.4%)	5.4% (+ 1.7%)	5.3% (+ 1.8%)	1.3% (+ 5.8%)
30%	13.1% (− 1.9%)	8.6% (+ 2.1%)	8.3% (+ 2.4%)	3.0% (+ 7.6%)
40%	16.6% (− 0.2%)	12.2% (+ 2.9%)	10.9% (+ 4.2%)	4.8% (+ 10.1%)
50%	20.9% (+ 0.7%)	17.4% (+ 2.3%)	14.9% (+ 4.8%)	6.7% (+ 13.0%)
60%	26.3% (+ 3.9%)	25.2% (+ 2.5%)	19.7% (+ 8.0%)	8.9% (+ 18.2%)
70%	33.1% (+ 8.7%)	33.9% (+ 4.4%)	27.8% (+ 10.5%)	12.3% (25.3%)
80%	42.3% (+ 11.4%)	44.6% (+ 5.1%)	36.0% (+ 13.4%)	17.9% (+ 30.5%)
90%	62.5% (+ 13.2%)	53.6% (+ 19.8%)	45.6% (+ 27.4%)	27.7% (+ 42.1%)
95%	75.6% (+ 42.6%)	71.1% (+ 38.3%)	51.1% (+ 58.2%)	39.6% (+ 55.3%)
100%	167.4% (+ 42.6%)	100.0% (+ 84.0%)	100.1% (+ 83.8%)	82.1% (+ 101.9%)
Number of levels	637	602	599	603

**Scenario b)**

We applied Statement #1 to calculate single BG from multiple IG signals of different compartments, using the a priori determined parameters of study^5^. The calculated BG exhibited less relative error than Scenario a) that used a single IG signal.

- See as Table 3 presents less relative error than "A priori determined parameters – Scenario a)" column of Table 2.

**Table 3:** Scenario b), calculated single BG using multiple IG signals.

Cumulative probability of less than or equal relative error	Relative error (and improvement over IG that is considered as BG)				
	A priori determined parameters				BG-personalized parameters
	Subcutaneous tissue and visceral fat	Skeletal muscle and visceral fat	Subcutaneous tissue and skeletal muscle	All IG signals	All IG signals
10%	4.0% (− 1.2%)	3.9% (− 0.5%)	2.6% (+ 1.0%)	2.8% (+ 0.5%)	0.9% (+ 2.3%)
20%	6.8% (− 0.3%)	6.3% (+ 0.2%)	4.6% (+ 3.1%)	5.5% (+ 1.4%)	1.7% (+ 5.3%)
30%	10.1% (+ 0.6%)	9.2% (+ 1.2%)	7.1% (+ 5.8%)	7.2% (+ 3.7%)	2.5% (+ 8.5%)
40%	13.4% (+ 1.3%)	11.6% (+ 2.2%)	10.3% (+ 7.8%)	9.6% (+ 5.5%)	3.9% (+ 11.4%)
50%	17.1% (+ 3.9%)	14.5% (+ 6.0%)	14.4% (+ 10.1%)	12.3% (+ 8.7%)	5.1% (+ 17.2%)
60%	21.2% (+ 8.9%)	17.6% (+ 12.6%)	18.5% (+ 12.1%)	16.1% (+ 13.7%)	6.3% (+ 23.7%)
70%	29.5% (+ 13.8%)	20.1% (+ 18.2%)	24.4% (+ 18.9%)	20.5% (+ 19.1%)	9.3% (+ 31.0%)
80%	39.7% (+ 13.8%)	22.7% (+ 24.7%)	32.5% (+ 24.5%)	28.1% (+ 21.8%)	13.2% (+ 38.4%)
90%	47.4% (+ 27.9%)	32.8% (+ 36.0%)	49.7% (+ 28.3%)	38.7% (+ 32.8%)	18.5% (+ 53.2%)
95%	53.2% (+ 50.7%)	44.4% (+ 38.7%)	59.4% (+ 61.5%)	44.4% (+ 59.1%)	23.8% (+ 80.1%)
100%	77.5% (+ 90.3%)	82.2% (+ 89.3%)	121.4% (+ 50.1%)	93.3% (+ 78.2%)	41.7% (+ 129.9%)
Number of levels	454	492	485	768	783

**Scenario c)**

With Statement #1, we calculated BG from multiple IG signals. With Statement #2, we determined personalized parameters of the model per each IG signal. Then, we calculated individual BG signals from each IG signal. When compared to the measured BG, Statement #2 exhibited less relative error than Scenario a).

- In Table 2, see as the "IG-only Statement #2-personalized parameters – Scenario c)" column reduces relative error over the "A priori determined parameters – Scenario a)" column.

**Scenario d)**

With Scenario c) the relative error is less than with Scenario a), but not necessarily with Scenario b), because Scenario c) evaluated individual, calculated BG signals with the a priori determined parameters of study^5^ as well as Scenario b). To reduce the relative error further, we repeatedly applied Statement #3. Eventually, we reduced the relative error when compared to the Scenario c). Specifically, we improved the precision, because the relative error is comparable to Scenario b) as it converged towards its (Scenario b) BG calculation.

- Compare Tables 3 and 4 to see, as the relative errors are comparable.
- In Table 2, see as the "IG-only Statement #3-personalized parameters – Scenario d)" column gives less relative error than the "IG-only Statement #2-personalized parameters – Scenario c)" column.

**Table 4:** Scenario d), calculated single BG using multiple IG signals and personalized parameters that were determined from IG signals only.

Cumulative probability of less than or equal relative error	Relative error (and improvement over IG that is considered as BG)			
	Subcutaneous tissue and visceral fat	Skeletal muscle and visceral fat	Subcutaneous tissue and skeletal muscle	All IG signals
10%	2.5% (+ 0.3%)	3.8% (− 0.4%)	2.5% (+ 1.1%)	3.1% (+ 0.2%)
20%	5.5% (+ 1.0%)	6.5% (− 0.2%)	4.9% (+ 3.2%)	5.2% (+ 1.8%)
30%	8.3% (+ 2.5%)	8.2% (+ 2.1%)	7.0% (+ 6.0%)	6.6% (+ 4.4%)
40%	12.3% (+ 2.6%)	10.9% (+ 2.9%)	10.5% (+ 8.2%)	9.3% (+ 6.2%)
50%	17.7% (+ 4.1%)	14.2% (+ 6.8%)	16.2% (+ 8.4%)	12.6% (+ 9.2%)
60%	24.8% (+ 5.5%)	17.0% (+ 13.5%)	18.8% (+ 12.6%)	16.8% (+ 13.1%)
70%	34.4% (+ 8.9%)	20.4% (+ 18.4%)	26.2% (+ 17.6%)	21.9% (+ 17.5%)
80%	41.9% (+ 11.8%)	23.2% (+ 24.6%)	35.5% (+ 23.6%)	32.5% (+ 17.4%)
90%	48.6% (+ 26.7%)	34.8% (+ 34.1%)	53.1% (+ 25.0%)	40.3% (+ 30.8%)
95%	55.0% (+ 48.9%)	45.5% (+ 45.4%)	58.1% (+ 62.7%)	43.6% (+ 55.4%)
100%	76.5% (+ 91.3%)	88.3% (+ 83.3%)	120.0% (+ 51.5%)	82.9% (+ 85.0)
Number of levels	458	496	493	778

Tables 2, 3 and 4 give empirical cumulative distribution function (ECDF) of relative error of calculated BG. ECDF represents the probability that the relative error of calculated BG is less than or equal a given relative error. We sorted the relative errors in ascending order.

Thus, we obtained the empirical distribution function, as a step function with a stepping of *1/n*. *n* is the number of measured IG, for which we calculated and measured BG. According to the Glivenko–Cantelli theorem, such an empirical distribution function converges to the true distribution function^21,22^. *n*-value depends on the *Δt*_*f*_ parameter^18^.

To determine model parameters, we used Meta-Differential Evolution as with study^5^. As the fitness function, we used sum of squared absolute differences between measured and calculated glucose levels. The Meta-Differential Evolution used the full precision of the IEEE 754 64-bit floating-point numbers. Nevertheless, we did not apply local search due to computational requirements and used the a priori determined parameters instead of the analytical method. Analytical method would require reference BG and hence we could not use it.

In Tables 2, 3 and 4, the less relative-error (i.e., the number before the rounded brackets) is better and the greater improvement over IG (i.e., the number in the rounded brackets) is better. Considering IG as sufficiently close to BG is current clinical practice, when examining CGMS records.

Table 2 analyzes relative error, when calculating BG with a single IG signal and a set of given parameters. Specifically, this table analyzes the error for Scenarios a), c) and d). In addition, it analyzes error of model parameters, which were determined using measured BG to estimate maximum possible accuracy.

Table 3 analyzes relative error of Scenario b), in which we calculated BG from multiple IG signals, using the a priori determined parameters. This table includes all combinations of the measured IG signals in different compartments. In addition, the last column of this table gives the estimated best possible accuracy of the proposed model. We estimated this accuracy by applying Statement #1 to individual BG signals, which we calculated with personalized model parameters. We determined the personalized parameters with measured BG per each individual compartment.

Table 4 analyzes relative error for Scenario d), in which we calculated BG from multiple IG signals of different compartments and determined personalized model parameters from these signals only. As with Table 3, this table includes all combinations of measured IG signals.

Table 1 summarizes model parameters for Table 2. It demonstrates as Statement #2 produced personalized parameters, by showing that they differ from the initially used, a priori determined parameters.

Table 5 summarizes the results with PEG as ISO 15197:2013 standard specifies this grid for evaluating the glucose monitoring systems^23^.

**Table 5:** Parkes’ error grid for diabetes type-1; calculating BG with All IG signals.

Scenario	Parkes’ error grid zone percentage of calculated glucose levels					
	A (%)	B (%)	A + B (%)	C (%)	D (%)	E (%)
Scenario b) with BG-personalized parameters (adaptive due to the calibration, BG measurements required)	93	7	100	0	0	0
Scenario b) with a priori determined parameters (non-adaptive, no BG calibration)	71	28	99	1	0	0
Scenario d) IG-only determined parameters (adaptive, no BG calibration)	70	30	100	0	0	0

Note as the Scenario d), with IG-only determined parameters, reduced the C-zone percentage as an improvement to the initial, a priori determined parameters – i.e., Scenario b), while having 100% of calculated levels in the clinically-safe zones.

**Figure 2:**
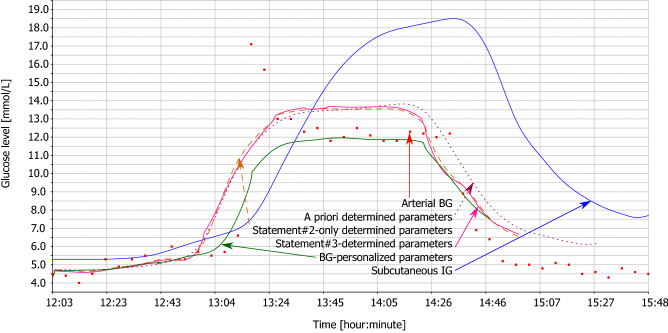
Animal #1 – BG calculation using multiple IG signals.

**Figure 3:**
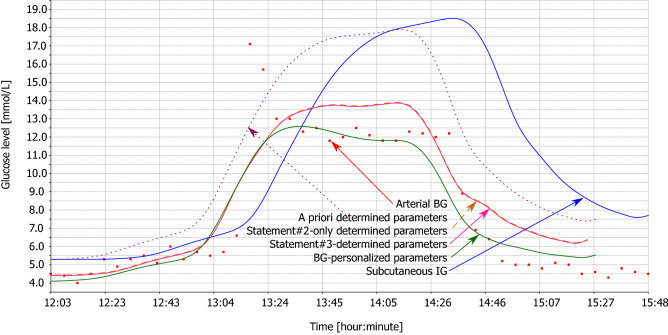
Animal #1 – BG calculation using individual IG signal.

**Figure 4:**
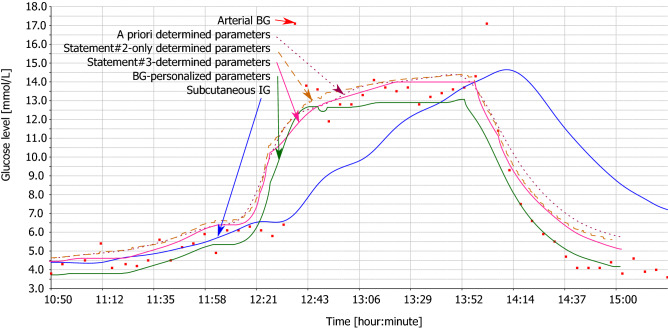
Animal #2 – BG calculation using multiple IG signals.

**Figure 5:**
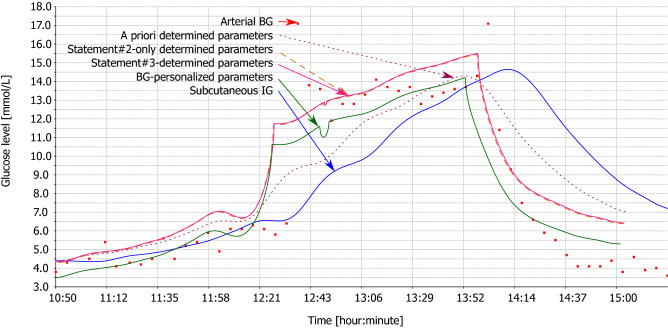
Animal #2 – BG calculation using individual IG signal.

Figures 2, 3, 4 and 5 illustrate the tables with two animals out of all animals of this study. Each animal comprises two figures, which depict calculated BG. While the first figure depicts BG calculation using multiple IG signals, the second figure depicts BG calculation from the subcutaneous tissue IG only, using the same set of parameters. This demonstrates the effect of individual statements. Red square denotes measured arterial BG. Solid blue curve denotes measured IG of subcutaneous tissue. Dotted wine curve represents Statement #1 with a priori determined parameters. Dashed brown curve represents single application of Statement #2. Solid pink curve represents Statement #3, i.e., repeated application of Statements #1 and #2. Solid green curve represents the estimated best possible accuracy.

For the sake of clarity of the figures, we plotted only the subcutaneous IG signal as this signal is measured with human patients with diabetes. Study^20^ discusses differences of the dynamics of individual IG signals in detail.

## Discussion

With the a priori determined parameters, the proposed model of glucose dynamics reduced relative errors greater than 15%, when compared to the CGMS output without applying any of the statements. In Table 2, the "A priori determined parameters – Scenario a)" column demonstrate this. Until the 15%, the a priori determined parameters exhibit decreased accuracy than the CGMS output. This is a result of eliminating the *h* and *k* parameters from the model. These parameters account for variable IG delay in a single IG signal^18^. It is a trade-off for avoiding the possible overfitting of the proposed model of glucose dynamics. Nevertheless, we consistently reduced the relative error at all probabilities by applying Statements #1 and #2 exactly once, i.e., Scenario c, with the a priori determined parameters. Statement #3 increased the accuracy further, by processing an individual IG signal with personalized parameters.

Due to the challenging experimental setup, calculated BG exhibits a certain error as described in Tables 1, 2and3. There are two major source of the errors. The first one is the rapid BG increase due to intra-venous glucose bolus, which produces a short-lived BG peaks. In accordance with studies^19,20^, we do not consider these peaks as important as the longer-lived BG patterns. Nevertheless, these peaks increase the overall resulting relative error. The second major source of the errors is the intravenously given insulin bolus. BG decreases considerably faster than glucose levels of the other compartments. As a result, calculated BG is greater than arterial BG. In a daily life of a common patient with diabetes, such intravenous boluses are not given and thus there are no such rapid changes. Therefore, we do not expect these particular error-sources to be a major issue once the research advances to human CGMS profiles.

Table 3 demonstrates that a priori determined parameters were sufficient to outperform CGMS at all probabilities of relative error, once we applied Statement #1. Most importantly, it significantly reduced relative errors, which exceeded 15%. Error grid analysis, such as Clarke Error Grid and Parkes Error Grid (PEG), comprises five zones, A–E, with increasing severity of relative error^23,24^. In zone E, hypoglycemia can be confused with hyperglycemia and vice versa. Therefore, it is important to reduce the maximum errors to move them to less dangerous zones – this was the effect of Statement #1.

Hyperglycemic clamp comprises two transitions: a transition from normal BG to hyperglycemic plateau and then back to normal BG. As depicted in Figs. 2, 3, 4, and 5, IG completely disagreed with BG since the first transition. At the first transition, the proposed method of BG calculation was only unable to reconstruct the arbitrarily invoked BG peak. This is likely due to biological reasons rather than technological reasons. Experimenter rapidly saturated blood with glucose, while there was no associated physiological glucose increase in the other compartments. At the second transition, calculated BG exhibited a small time-lag behind the arterial BG. This is a result of eliminating the *k* and *h* parameters from the original model^18^ – see Eq. (1). Nevertheless, the figures depict as Statement #2 reduced this lag, when compared to Statement #1 with the a priori determined parameters. Yet, the figures confirm that Statement #1 with a priori determined parameters was sufficient to outperform IG when estimating BG.

Table 5 demonstrates that BG-calibration considerably reduces the BG-calculation error. Nevertheless, this approach presents an important discomfort to the patient. If we would use a priori determined parameters only, then some of the calculated BG levels could fall outside the clinically safe A + B zones. If we would continuously adapt the parameters from the IG-signals only, then the results suggest the possibility of avoiding the dangerous zones.

Wearing three different sensors would be challenging for the patient. Nevertheless, we are proving the concept at this phase of our research. We prove that it is possible to calculate BG from three different IG signals. A particular solution of obtaining three different signals is a subject to a future research, perhaps on a novel sensor design. Such a design could also incorporate a specific error model of the sensor, possibly utilizing additional physiological signals to reduce the dynamic error-variability due to changing metabolic needs^25^.

## Conclusion

We have demonstrated that multiple sensors improved accuracy of calculated BG. Therefore, we would like to initiate a research on a use of multiple sensors for patients with diabetes, whose BG is not well stabilized and shows considerable, irregular peaks. In the clinical practice, when BG is measured and considered suspicious (e.g., too much elevated), the measurement repeats after several minutes. If a physician considers the second measured BG as close to the true BG, the second measurement is accepted and the first one discarded^26^. There is no verification whether the first reading was correct or not. Let us stress the possibility that BG can experience rapid peak, which may disappear while waiting to make the second measurement. As a result, important information about the diseases could be lost. Our method could reduce such an uncertainty about measurement correctness.

We have also demonstrated that it is possible to determine personalized parameters from IG signals only. Looking at the progress of fault-tolerant systems (e.g., in the aerospace industry), which process multiple signals (IG in our case) to achieve an agreement (BG in our case), we believe that it is possible to develop an autonomous CGMS-calibration protocol. That would prolong sensor’s lifetime, while adapting to recent physiological changes and metabolic processes of a specific patient without requiring the patient to draw any drop of blood to calibrate the sensor. With this study, we hope to start this development. It would benefit the patients with diabetes by making the CGMS just a long-term, perhaps an implantable device^13^ that needs no additional care^12^. Increased acceptance of such a user-friendly CGMS would lead to improved treatment, thus reducing the costs incurred by inadequately compensated BG.

Eventually, this work would benefit the development of artificial pancreas, i.e., a closed-loop system, which presently doses insulin based on CGMS-measured IG at a single location.

In the real life, a patient with diabetes experience both hypo- and hyperglycemic glucose levels. In this paper, we addressed normo- and hyperglycemic glucose levels. Therefore, our future research needs to validate the proposed approach with a hypoglycemic scenario.
